# 522 A Single Institution’s Recent Experience with Deep Partial-Thickness Friction Burns

**DOI:** 10.1093/jbcr/iraf019.151

**Published:** 2025-04-01

**Authors:** Thomas Vogler, Bethany Schmoker, Arek Wiktor, Cameron Gibson

**Affiliations:** University of Colorado; University of Colorado Hospital; University of Colorado; University of Colorado

## Abstract

**Introduction:**

Friction burn depth is often variable, ranging from superficial to full thickness. The standard of care (SOC) for full-thickness friction burns often includes autologous split-thickness skin grafts. However, the SOC for deep partial-thickness or indeterminate depth friction burns is less clear. Identifying the optimal surgical treatment approach to these wounds may help improve healing times and decrease wound care needs for patients. We therefore sought to summarize our experience managing deep partial thickness friction burns.

**Methods:**

We performed a retrospective review of all patients with friction burns admitted to our ABA verified burn center from 2016-2024. We included patients with (1) deep partial-thickness friction burns requiring non-tangential excision, (2) contiguous wounds greater or equal to 1% total body surface area (TBSA), who (3) were admitted to Burn Surgery as the primary service, (4) had secondary injuries no greater than an Abbreviated Injury Scale of 2 and (5) had consistent follow up with a Burn provider.

**Results:**

Of the 56 patients examined, 14 patients met these criteria with a total of 26 separate wounds greater than 1% TBSA. Of the 26 wounds included, 14 were treated using non-tangential excision alone and local wound care. Twelve were treated using non-tangential excision with epidermal autograft or a biologic dressing. At 4 weeks post injury, significantly fewer of the wounds in the excision alone group were healed (57%, 8/14) versus the wounds in the epidermal autograft/biologic dressing group (100%, 12/12) (Fischer Exact Test = 0.02). The healing time was more highly variable with a trend towards increased overall healing time in the excision alone group (28.5 +/- 14 days; Median: 24) compared to the epidermal autograft/biologic dressing group (20.7 +/- 5 days; Median: 21) (Wilcoxon Rank Sum p = 0.1).

**Conclusions:**

These data suggest using epidermal autograft or biologic dressing on deep partial-thickness friction burns provides a potentially more consistent, uniform healing time compared to excision and local wound care, however more data is needed to validate this finding. Further work is also needed with long-term follow-up to examine differences in scar formation, functionality and cosmetic outcomes in each group.

**Applicability of Research to Practice:**

When evaluating treatment options for deep partial-thickness friction burns, wound closure rates will be significantly higher at four weeks post injury if epidermal autograft or biologic dressings are used compared to excision and local wound care alone. The long-term differences between these two groups are less clear and need further analysis.

**Funding for the Study:**

N/A

